# Chemical and physical interactions of regenerated cellulose yarns and isocyanate-based matrix systems

**DOI:** 10.1038/s41598-021-91115-4

**Published:** 2021-06-02

**Authors:** Bernhard Ungerer, Ulrich Müller, Antje Potthast, Enrique Herrero Acero, Stefan Veigel

**Affiliations:** 1 Department of Material Sciences and Process Engineering, Institute of Wood Technology and Renewable Materials, University of Natural Resources and Life Sciences, Konrad-Lorenz-Straße 24, 3430 Vienna, Tulln Austria; 2 Department of Chemistry, Institute of Chemistry of Renewable Resources, University of Natural Resources and Life Sciences, Konrad-Lorenz-Straße 24, 3430 Vienna, Tulln Austria; 3 Glanzstoff Management GmbH, Technopark 1C Top 17, 3430 Tulln, Austria

**Keywords:** Biopolymers, Composites, Mechanical properties

## Abstract

In the development of structural composites based on regenerated cellulose filaments, the physical and chemical interactions at the fibre-matrix interphase need to be fully understood. In the present study, continuous yarns and filaments of viscose (rayon) were treated with either polymeric diphenylmethane diisocyanate (pMDI) or a pMDI-based hardener for polyurethane resins. The effect of isocyanate treatment on mechanical yarn properties was evaluated in tensile tests. A significant decrease in tensile modulus, tensile force and elongation at break was found for treated samples. As revealed by size exclusion chromatography, isocyanate treatment resulted in a significantly reduced molecular weight of cellulose, presumably owing to hydrolytic cleavage caused by hydrochloric acid occurring as an impurity in pMDI. Yarn twist, fibre moisture content and, most significantly, the chemical composition of the isocyanate matrix were identified as critical process parameters strongly affecting the extent of reduction in mechanical performance. To cope with the problem of degradative reactions an additional step using calcium carbonate to trap hydrogen ions is proposed.

## Introduction

The increasing importance of environmental considerations in materials engineering has turned the intention of research and industry to minimising energy demand and the ecological impact of new products. In the case of food packaging, for example, numerous renewables- or recycling-based product solutions have already become established^1,2^. The feasibility of complex applications for structural elements, however, has not been proven: can materials from renewable sources also be applied in composites designed for high mechanical loads? The feasibility of bio-based composites in structural applications has already been tested in the automotive industry, using composites based on engineered wood products^3–5^.

In high-performance and lightweight composites, continuous filaments typically constitute the reinforcement component^6–8^. For this purpose, glass, carbon and, to a lesser extent, synthetic polymers such as aramid are normally used^9^. In responding to the question above, first, renewable materials that are able to compete with these current sources have to be detected. The main technical criteria for substitution are the fulfilment of mechanical thresholds, the predictability of material properties, flexibility in terms of product dimensions, as well as availability.

A bio-based source that may meet these requirements is regenerated cellulose. Obtained from cellulose pulp by different spinning processes, vast availability is given^10,11^. When processed as continuous filaments, dimensional flexibility is afforded as well. Depending on their molecular weight and crystallinity, cellulose fibrils and fibres possess high specific tensile strength and tensile modulus^12,13^, making them widely competitive with synthetic fibre materials in terms of mechanics. With respect to a broader application as fibre reinforcement, however, the predictability of cellulosic material properties remains a challenge.

In contrast to non-polar carbon or polyester fibres, cellulose, thanks to its molecular structure, which features a surface stacked with hydroxyl (OH) groups, is susceptible to oxidative or substitution reactions^14^, as well as hygroscopic phenomena^15^. Moreover, hydroxyl groups are potential reactants in the curing process of thermosetting resins such as epoxides and polyurethanes, which are widely used in the field of composite structures. For this reason, a systematic understanding of how cellulose filaments interact on a physical and chemical level with each relevant matrix material needs to be acquired. In the present work, investigations focus on isocyanates (R-NCO) as a precursor material used in frequently applied matrix and adhesive systems such as polyurethane (PUR), emulsion polymer isocyanate (EPI) or polymethylene polyphenyl isocyanate (pMDI)^16,17^. In the context of wood adhesion and modification, interactions between isocyanates and cellulosic matter have been extensively studied^18^. Whereas older studies ascribed strong adhesion and altered surface properties to reactions between wood–OH and matrix–NCO groups^19^, more recent findings suggest that such covalent bonds are unlikely to occur^20^. Instead, low molecular-weight matrix substrate could infiltrate the cell wall^21^, leading to mechanical interlocking, hydrogen bonding and van der Waals interactions with the cell wall^22^.

To the authors’ knowledge there have been no reports to date on the mechanical effects of isocyanate compounds on regenerated cellulose filaments. Technical viscose from tyre cord fabrication has served as source of cellulose. Samples from fabric yarns down to single filaments have been analysed to capture a possible size effect. As a source of isocyanate, the hardener of a two-component polyurethane resin, applied on an industrial scale for wood adhesion, as well as a laboratory-grade pMDI reference have been used. In relation to the extensive discussion on the plausibility of reactions between isocyanate and wood substrate, the implications that such covalent modifications could have on the mechanical performance of continuous cellulose filaments remain unknown. Rather than focusing exclusively on the possibility and special conditions under which such covalent modifications could occur^20^, the present study aims to give an overview on the physical and chemical effects, pMDI-based substances may have on cellulose when applied as matrix component. This includes furthermore side effects from impurities such as HCl, a by-product inevitably arising during isocyanate synthesis^23^. To this date, the impact of acidity on cellulose within a non-polar medium remains a scarcely investigated issue.

From a general point of view, it is to be anticipated that the adhesion between isocyanate and cellulose is the result of attracting forces caused by physical and chemical interactions. However, the direct quantification of individual interaction effects via measures of analytical chemistry is extremely challenging. As a first stage, it may thus be useful to focus on measuring the mechanical implications of cellulose–isocyanate interactions. This may allow indirect evidence to be collected for both physical and chemical processes on the macro- and the nanoscale. As a second step, a set of chemical explanations for interactions observed may be offered by means of Fourier-transform infrared spectroscopy (FTIR) and size exclusion chromatography combined with multi-angle laser light scattering (SEC–MALLS).

As a principal hypothesis, the decrease in mechanical performance and altered molecular structure of cellulose filaments as a consequence of interaction with isocyanate-based substances is verified. More specifically, the significant impact of acid impurities on cellulose within a non-aqueous medium is assessed.

## Materials and methods

### Materials

Tyre cord viscose was used to study interactions with isocyanate compounds. All viscose yarns were provided by Glanzstoff Bohemia Ltd (Lovosice, Czech Republic). Yarns with titres of 1840 dtex and 2440 dtex (1 dtex ≙ 1 g/10000 m), both with and without surface finishing (avivage), were tested. The 2440 dtex samples carrying avivage were obtained from a fabric with a 1/1 plain weave structure, permitting warp and weft yarns to be examined separately. Yarns obtained from the fabric additionally featured a Z100 twist (i.e. 100 twists per metre). All yarn samples were stored under standard conditions of 20 °C and 65% relative humidity (RH). The preparation of single filament samples was done according to Adusumalli et al.^24^, requiring paper frames for adjusting the samples, on to which the viscose filaments were glued with a droplet of instant adhesive. Before and after the isocyanate treatment, samples were stored under standard conditions of 20 °C and 65% RH. For the isocyanate treatment described below, all cellulose samples were conditioned either at 20 °C, 65% RH for a minimum of 48 h or oven-dried for a minimum of 24 h at 103 °C.

### Isocyanate treatment

In order to study interactions between cellulose and isocyanates under conditions relevant for the production of engineered wood products and composite structures in general, the hardener of a polyurethane resin (RP 3007), provided by Collano (Sempach, Switzerland), was used. The hardener consists of isomers and homologues of polymeric diphenylmethane diisocyanate (pMDI, CAS-No. 9016-87-9) and diphenylmethane diisocyanate (MDI, CAS-No. 101-68-8, 2536-05-2 and 5873-54-1). pMDI with average ${\text{M}}_{\text{n}}$ ~ 340 g/mol (CAS-No. 9016-87-9, SIGMA-ALDRICH, Schnelldorf, Germany) was used as a reference agent for the yarn treatment. The conditioned fibre samples (either 20 °C, 65% RH or 103 °C kiln-dried) were completely immersed in isocyanate and stored under laboratory conditions (approximately 20 °C, 50% RH). The duration of immersion ranged from a couple of seconds for some of the filament samples to 15 days for a long-term treatment, in order to observe a possible time dependency. Treatment times for each batch are listed in Table 1. After the isocyanate bath, samples were removed, excess dipping agent was skimmed off with cleaning paper and all samples were folded into three layers of cleaning paper and stored for drying under standard conditions of 20 °C and 65% RH. The amount of isocyanate agent remaining on the yarn was determined by comparing yarn sample mass before and after the isocyanate impregnation. The mass of untreated yarns was directly derived from their linear density (dtex), while, in the case of treated yarns, 10 samples of defined length (100 mm) per batch were weighed on an analytical balance and the matrix percentage was calculated according to Eq. (1).

**Table 1:** Processing conditions for the preparation of isocyanate-treated viscose yarns and results from tensile tests (mean values ± standard deviation).

Batch	dtex	Orientation	Avivage	Fibre condition at treatment	Dipping time (days)	Amount matrix (%)	Sample size	Maximum load (N)	Tenacity (cN/tex)	Elongation at break (%)	Tensile modulus (GPa)
ref 1	2440^a^	Twisted	Yes	–	–	–	15	100.0 ± 3.2	4.3 ± 0.3	12.6 ± 0.6	14.6 ± 0.6
ref 2	2440^b^	Twisted	Yes	–	–	–	20	102.7 ± 1.9	4.2 ± 0.1	13.7 ± 0.5	14.3 ± 0.5
ref 3	2440	Untwisted	No	–	–	–	20	104.7 ± 7.4	4.1 ± 0.1	11.6 ± 1.1	16.3 ± 0.9
iso 1	2440^a^	Twisted	Yes	20 °C, 65%RH	4	68.2	7	32.6 ± 3.8	1.3 ± 0.2	2.7 ± 0.3	10.7 ± 2.1
iso 2	2440^b^	Twisted	Yes	20 °C, 65%RH	4	54.6	10	27.4 ± 6.4	1.1 ± 0.3	2.2 ± 0.5	8.5 ± 2.2
iso 3	2440	Untwisted	No	20 °C, 65%RH	4	54.4	9	35.9 ± 10.5	1.5 ± 0.4	2.9 ± 1.2	11.6 ± 2.4
iso 4	2440^b^	Twisted	Yes	20 °C, 65%RH	15	68.6	9	13.5 ± 15.5	0.6 ± 0.6	3.6 ± 2.8	2.4 ± 2.6
iso 5	1840	Untwisted	Yes	20 °C, 65%RH	4	49.1	6	27.7 ± 0.7	1.5 ± 0.0	3.0 ± 0.5	13.2 ± 1.1
iso 6	1840	Untwisted	No	20 °C, 65%RH	4	50.3	9	26.0 ± 4.0	1.4 ± 0.2	3.1 ± 0.7	12.0 ± 2.7
dry-iso 1	2440^a^	Twisted	Yes	103 °C, dry	4	37.3	10	56.4 ± 8.5	2.3 ± 0.3	9.1 ± 2.3	9.2 ± 1.1
dry-iso 2	1840	Untwisted	No	103 °C, dry	4	77.3	10	32.8 ± 10.8	1.8 ± 0.6	2.6 ± 1.5	14.9 ± 3.5
dry-iso 3	1840	Untwisted	Yes	103 °C, dry	4	53.0	7	39.2 ± 11.1	2.1 ± 0.6	6.4 ± 3.2	11.9 ± 2.2
pMDI 1	2440^a^	Twisted	Yes	20 °C, 65%RH	5	69.6	7	33.5 ± 4.5	1.4 ± 0.2	2.6 ± 0.4	10.1 ± 1.4
pMDI 2	2440	Untwisted	No	20 °C, 65%RH	5	–	10	64.0 ± 5.6	2.6 ± 0.2	9.4 ± 0.9	14.1 ± 2.5
fil-ref	1.8	–	Yes	20 °C, 65%RH	–	–	9	75.7 ± 8.0^c^	4.2 ± 0.4	13.4 ± 2.8	16.3 ± 0.8
fil-iso 1	1.8	–	Yes	20 °C, 65%RH	0	–	10	74.6 ± 1.8^c^	4.1 ± 0.1	13.7 ± 1.4	18.9 ± 1.4
fil-iso 2	1.8	–	Yes	20 °C, 65%RH	3	–	10	49.4 ± 6.7^c^	2.7 ± 0.4	6.6 ± 1.5	24.2 ± 4.2
fil-iso 3^d^	1.8	–	Yes	20 °C, 65%RH	7	–	15	48.7 ± 8.9^c^	2.7 ± 0.5	11.4 ± 1.3	16.3 ± 1.8
fil-iso 4^e^	1.8	–	Yes	20 °C, 65%RH	7	–	7	61.1 ± 6.6^c^	3.4 ± 0.4	20.5 ± 5.6	12.6 ± 1.1

^a^Warp yarn from fabric.

^b^Weft yarn from fabric.

^c^Displayed unit mN.

^d^NMP purified.

^e^iso + CaCO_3_, NMP purified.

1 $$\frac{{m}_{sample}- {m}_{pure \; yarn}}{{m}_{sample}}\times 100=wt\% isocyanate \;compound$$

**Figure 1:**
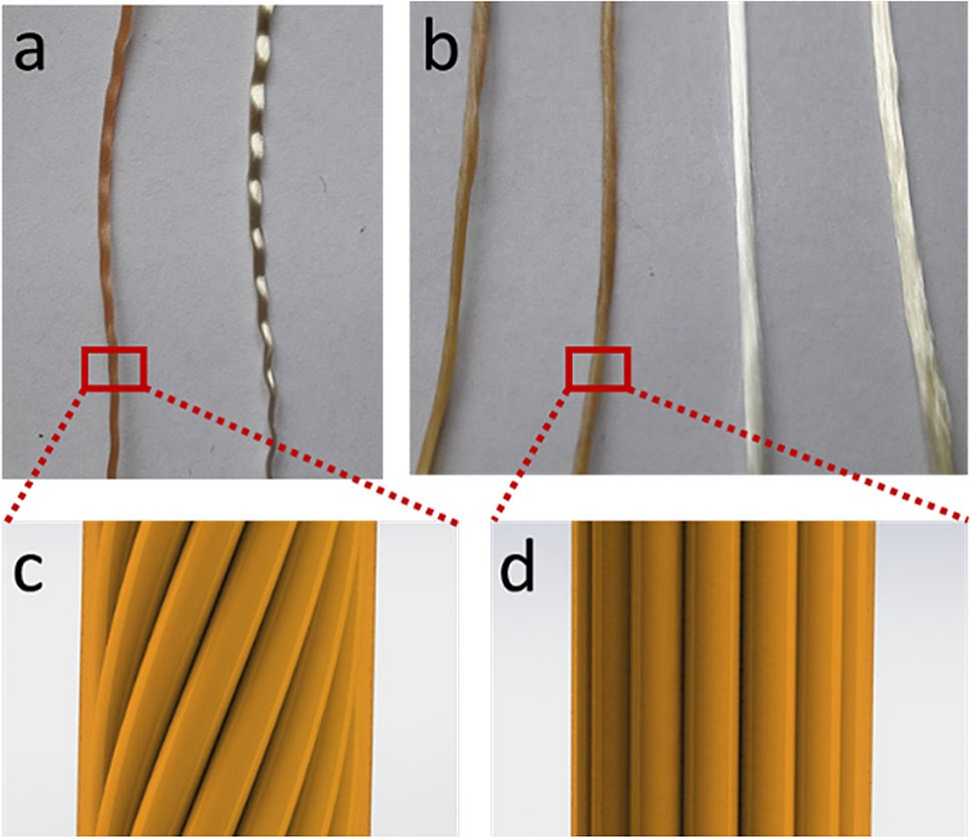
(**a**) 2440dtex twisted yarn iso (left) and ref (right) taken from fabric, (**b**) 2440dtex untwisted iso without avivage, iso with avivage, ref without avivage, ref with avivage (left to right), (**c**) schematic (!) of twisted, treated yarn (**d**) schematic (!) of untwisted treated yarn. Image c and d were drawn in SOLIDWORKS® 2019 SP3.0. Software available at https://www.solidworks.com/.

All the batches carrying isocyanate hardener were labelled as *iso*. All the batches carrying pMDI were labelled as *pMDI*. Untreated reference batches were labelled as *ref*. An overview of the different sample types is shown in Fig. 1.

### Mechanical characterisation

The mechanical properties of treated and untreated yarns and filaments were measured by means of tensile tests on a universal testing machine (Z020, Zwick/Roell, Ulm, Germany). The test software used was testXpert II. For untreated reference yarn samples (1840 and 2440 dtex), pneumatic yarn tensile grips from Instron (Nordwood, MA, USA) were used. An adapter was inserted to connect the Zwick and Instron systems. Specimen deformation was measured via crosshead displacement and a 1 kN load cell measured tensile force. Test speed was set to 25 mm/min, the range of tensile modulus evaluation was set between 10 and 20% of maximum load and the end of testing was defined as a 99% drop in force. Treated yarn samples, owing to their tendency towards embrittlement, could not be placed in the curved yarn channel of the pneumatic grips without leading to visible cracks on the sample surface. Therefore, the straight specimens were fixed with gummed clamps. Deformation was measured via crosshead displacement and tensile force was detected with a 500 N load cell. Test speed was set to 5 mm/min, the range of tensile modulus evaluation was set between 10 and 20% of maximum load and the end of testing was defined as a 99% drop in force. Treated and untreated filament samples were also fixed in gummed clamps. A load cell of 50 N was applied and deformation was measured via crosshead displacement. Test speed was set to 1.5 mm/min, the range of tensile modulus evaluation was set between 20 and 25% of maximum load and the end of testing was defined as a 95% drop in force. Before starting the measurement, the fixed paper frame carrying the single filament was cut on both sides, according to Adusumalli et al.^25^. This procedure should ensure straight alignment of the specimen. Regarding statistical evaluation of the mechanical tests, two sample t-tests at different variances were conducted and box and whisker charts (Microsoft Excel 2019, co. Microsoft, Redmond, WA, USA) were created.

In order to compare yarns/filaments of different linear density and structure in terms of stiffness, the tensile modulus was calculated by relating the load to the cross-sectional area of the respective yarn/filament. Specimen cross section can be determined according to Eq. (2), where *A* represents the cross section in mm^2^, *λ* the linear density in dtex (g/10^4^ m) and *ρ* the density in g/cm^3^. Tensile modulus was related to the cross section of viscose filaments only, i.e. a possible increase of the cross-sectional area due to isocyanate treatment was not taken into consideration.

2 $$A = \frac{\lambda }{\rho } \times {10}^{-4}$$

### Microscopic analysis

For qualitative observation of filament wettability, the digital microscope DSX1000 (co. Olympus, Tokyo, Japan) was used. An objective lens (DSX10-XLOB20X) with a 20 mm working distance and scanning mode polarisation observation was selected. Images were taken with a total magnification of 700.

### Chemical analysis

In order to test assumptions of what is happens on a chemical level between viscose and pMDI-based compounds, additional yarns treated with pMDI hardener were prepared. In contrast to the method described above, 2440 dtex samples (seven yarns of about 200 mm each) were immersed for seven days in a 150 ml beaker containing approximately 100 g of pMDI hardener. Instead of subsequent drying and curing, the treated samples were taken from the pMDI bath, excess liquid was skimmed off with cleaning paper and the yarns were immersed in a 150 ml beaker with approximately 50 ml of N-Methyl-2-pyrrolidone (NMP) (≥ 99%; SIGMA-ALDRICH, Schnelldorf, Germany), which is a well-established solvent for isocyanates and polyurethanes^26^. NMP was exchanged four times, with intervals of three, 12 and three hours between the cycles. Purified yarns were thoroughly rinsed in deionised water, folded into cleaning paper and stored at 20 °C, 65% RH. This procedure was repeated, the only difference being the addition of 012 g of calcium carbonate (CaCO_3,_ 98.5%; ROTH, Karlsruhe, Germany) to the pMDI hardener and thorough stirring of the mixture. Single-filament samples were prepared as described above in order to perform tensile tests.

Infrared measurements were conducted on a FTIR spectrometer (Frontier Universal ATR; Perkin Elmer, MA, USA), using the supplier’s software, Perkin Elmer Spectrum. For scanning, single 2440 dtex yarns of untreated viscose and viscose, following the above described procedure with NMP (without calcium carbonate), were placed in straight alignment on the ATR-crystal. The scanning range was set to 650–4000/cm, running 10 iterations per measurement. In order to facilitate a qualitative comparison, the spectra were normalised at 1000/cm.

SEC–MALLS for analysis of molar mass distribution was performed, according to Potthast et al.^27^. Two samples of untreated 2440 dtex viscose yarn and two samples of the above described procedure with NMP (without calcium carbonate) were measured.

## Results and discussion

### Mechanical characterisation of viscose yarns

In order to gain an overview of the most prominent parameters affecting the interaction between cellulose yarns/filaments and isocyanate-based matrix systems, mechanical tests were performed, employing a hands-on approach that was easily quantifiable. In the different batches of samples prepared (Table 1), the three main parameters analysed were the kind of isocyanate matrix applied, the fibre structure and the treatment conditions. The mechanical performance of a sample reflects the sum of all material parameters present. The following stepwise analysis is intended to disentangle this mixture of effects in the hope of reaching conclusions on how sample treatment affects material properties. The mechanical parameters used for comparison were maximum tensile load (N), elongation at break (%), and tensile modulus. In addition to tensile load, the respective tenacity values are given to facilitate the comparison between the different sample types. All samples with specimen-failure in the clamping area (rubber and pneumatic clamps) were excluded from evaluation. Where not otherwise stated, yarn treatment, storage and testing were conducted under 20 °C, 65% RH. All boxplots presented in this section consist of median, first and third quartile as well as minimum and maximum values (whiskers).

### Effect of isocyanate type and immersion time

**Figure 2:**
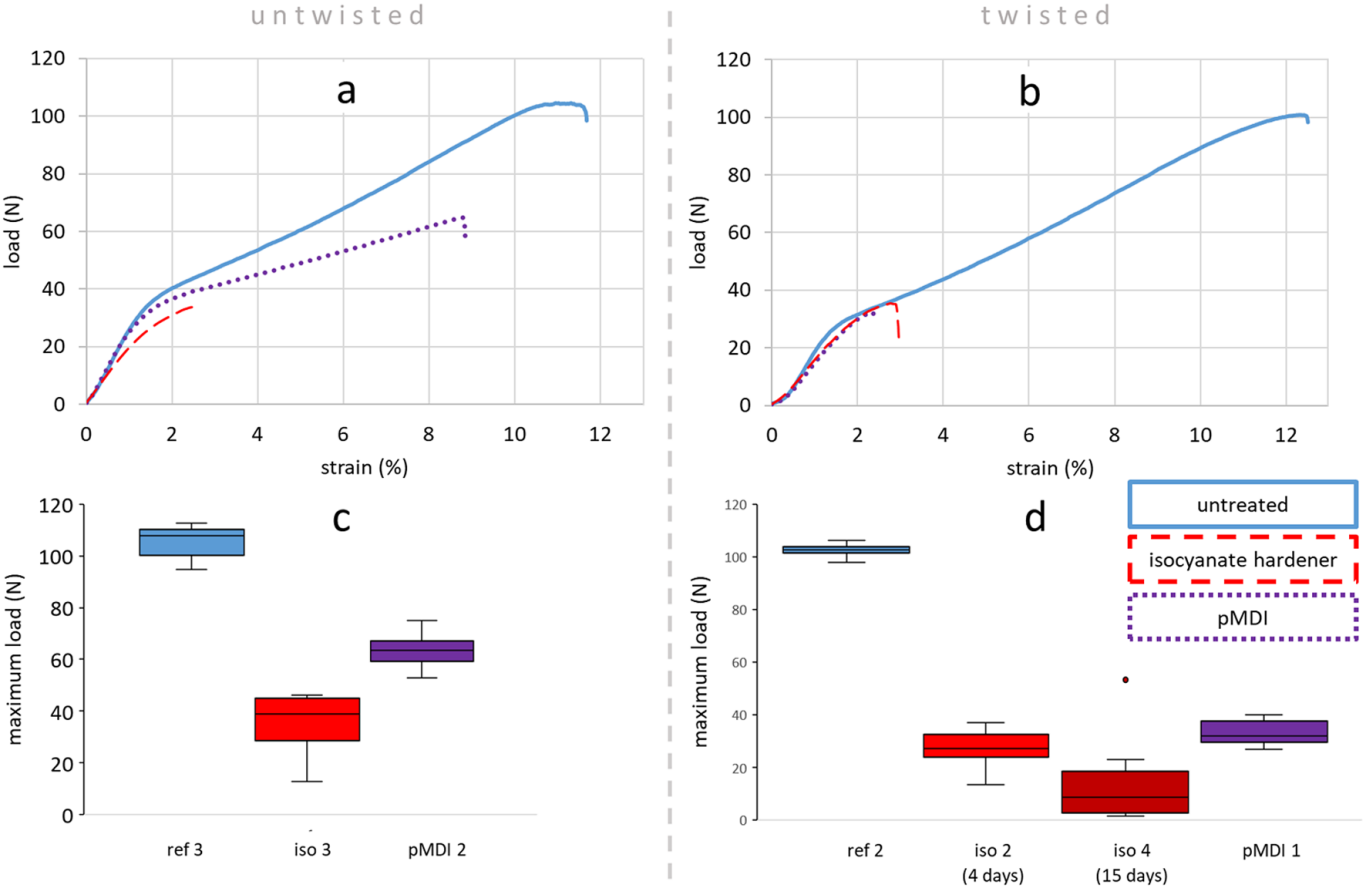
Tensile tests on 2440 dtex yarns; representative load-strain curves and maximum tensile load for untwisted (**a**, **c**) and twisted (**b**, **d**) samples, respectively. Immersion time is given in parenthesis (**d**).

Clear indications of a change in material properties of dipped filament yarns were observed. Figure 2a displays the force–strain diagram of untwisted yarns. The three curves represent the basic sample types: untreated viscose yarns as a reference (ref) and yarns either coated with isocyanate hardener (iso) or pure pMDI. Both treatments resulted in a significant decrease in tensile and elongation capability, the isocyanate hardener-dipped samples being even more severely affected. Those specimens closest to the mean tensile and elongation value of each batch are displayed in the graph. Compared to the untreated reference yarn, there is an overall loss in maximum tensile load of 66% for isocyanate hardener and 40% for pMDI samples, based on the mean values of each batch (see Fig. 2c). Elongation at break decreased by around 75% for isocyanate hardener and 22% for pMDI.

**Figure 3:**
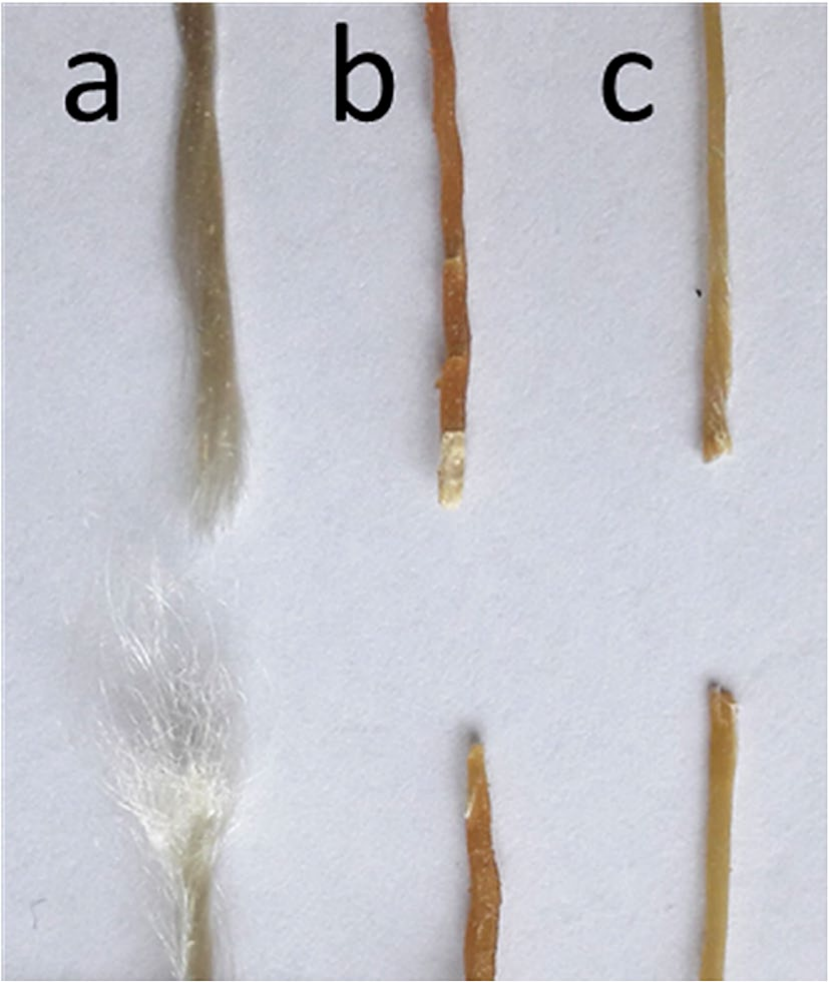
Characteristic fracture pattern of 2440dtex twisted yarn; untreated (**a**), isocyanate hardener (**b**) and pMDI (**c**) treated.

Figure 2b,d show the same evaluation for twisted yarns of 2440 dtex (Z100 drill = 100 twists per metre). The characteristic fracture pattern of these batches is shown in Fig. 3, indicating a tendency towards embrittlement for the treated samples. Whereas reference and isocyanate-hardener treated samples performed equally to the untwisted ones, pMDI samples featured lower tensile force and elongation at break, approximating the level of isocyanate hardener treated samples. For the hardener treated samples, in addition to the batch dipped for 4 days (iso 2) another with 15 days of dipping (iso 4) was added in order to observe an influence of treatment time. There was a significant difference between the two batches with the 15 days samples only reaching about half of the tensile force at break. Due to their brittle behaviour those samples had to be handled with care in order to avoid damages caused by clamping or excessive bending. As for the significant difference between isocyanate treated samples compared to untreated yarns, the additional decrease as a matter of longer treatment time could be an indication for chemical reactions between the cellulose and isocyanate compounds.

**Figure 4:**
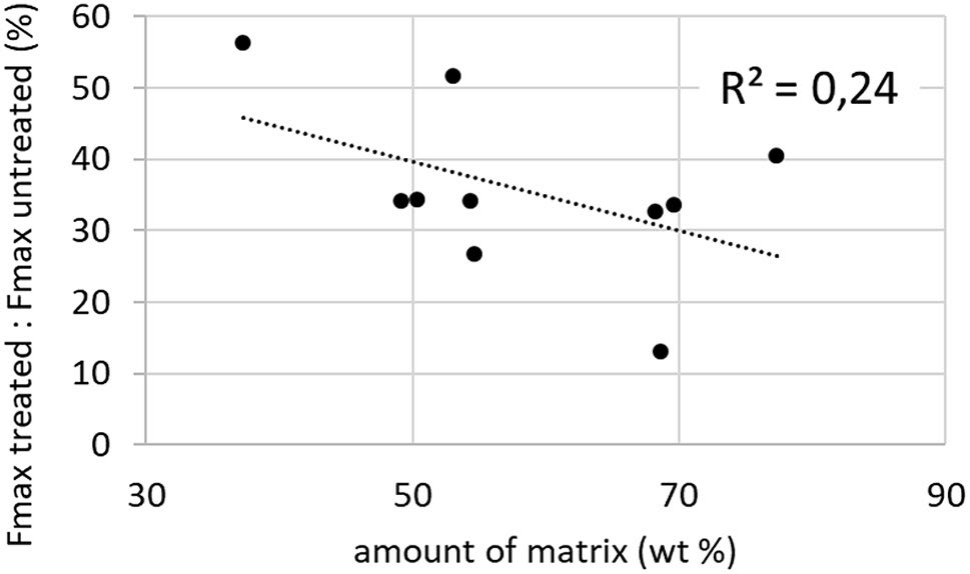
Ratio of tensile force at break of treated yarns and their untreated references over the average amount of isocyanate substance for each batch.

The one outlier in Fig. 2d represents a specimen which appeared to have less hardener on it and a paler hue, respectively. This observation might indicate a correlation between the amount of matrix and the mechanical performance as was verified in Fig. 4.

Each point in the graph represents one batch of samples. The ratio of average tensile force at break of treated to untreated yarns (y-axis) over the average amount of isocyanate substance incorporated within each batch (x-axis) are mapped. The reason for displaying a ratio instead of absolute values is the direct comparison of 1840 and 2440 dtex within one graph. Despite a low sample size (10 different batches) for statistical evidence by means of linear regression a tendency towards lower performance at higher matrix amounts can be observed with a fairly low coefficient of determination (R^2^) of 0.24.

Different reasons for the strong influence of isocyanate-based matrices on regenerated cellulose yarn are possible. On the one hand, chemical interactions are likely to occur. Evidence for such effects, however, has been reported to date only for wood–isocyanate interactions under restricted conditions^28^, as mentioned above. On the other hand, physical phenomena can have a negative effect. When incorporated into a stiff matrix, filaments within a yarn are subjected to local stresses. Furthermore, it is well known from textile technology that the performance of a yarn decreases with rising crimp angle, i.e. the degree of deviation from a straight orientation^29^.

### Effect of yarn twist

**Figure 5:**
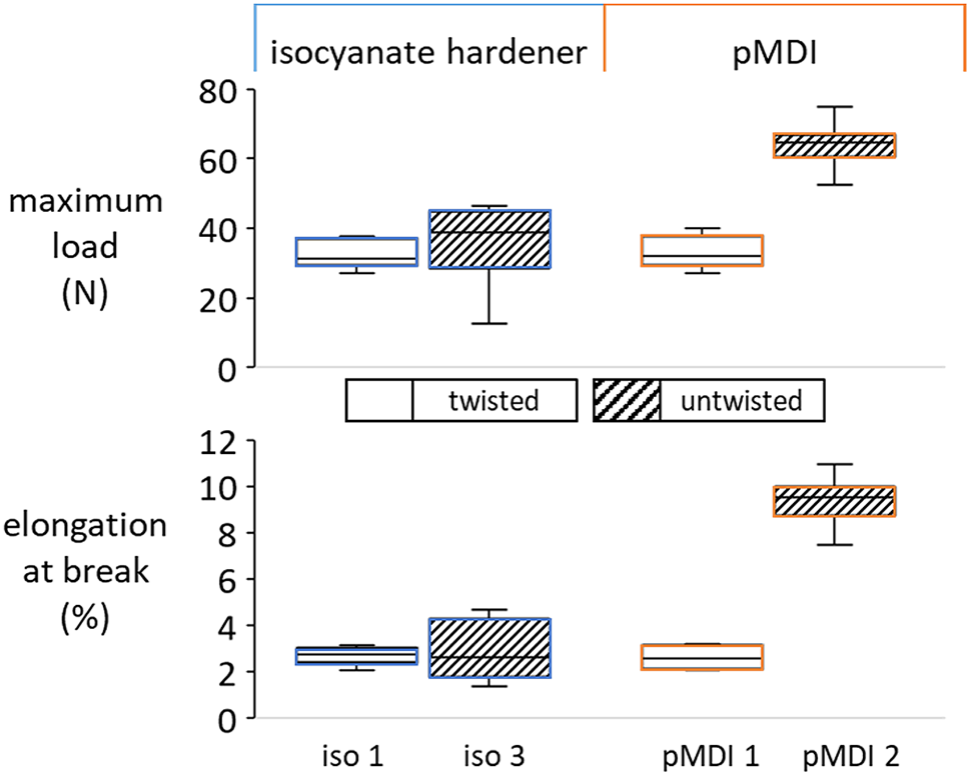
Maximum tensile load and elongation at break of twisted (white) and untwisted (grey) viscose yarns treated with isocyanate hardener (iso) and pMDI, respectively.

In order to analyse physical effects arising from the yarn structure, straight and twisted samples of 2440 dtex are compared in Fig. 5. Whereas for the isocyanate-hardener samples no significant difference between twisted and untwisted can be detected, pMDI samples with straight fibre alignment had about 48% and 52% higher maximum tensile load and elongation at break, respectively (based on mean values). The parameter of fibre orientation for pMDI treatment therefore appears to be a crucial one. For samples treated with isocyanate hardener, however, another factor seems to overlap these physical ones.

**Figure 6:**
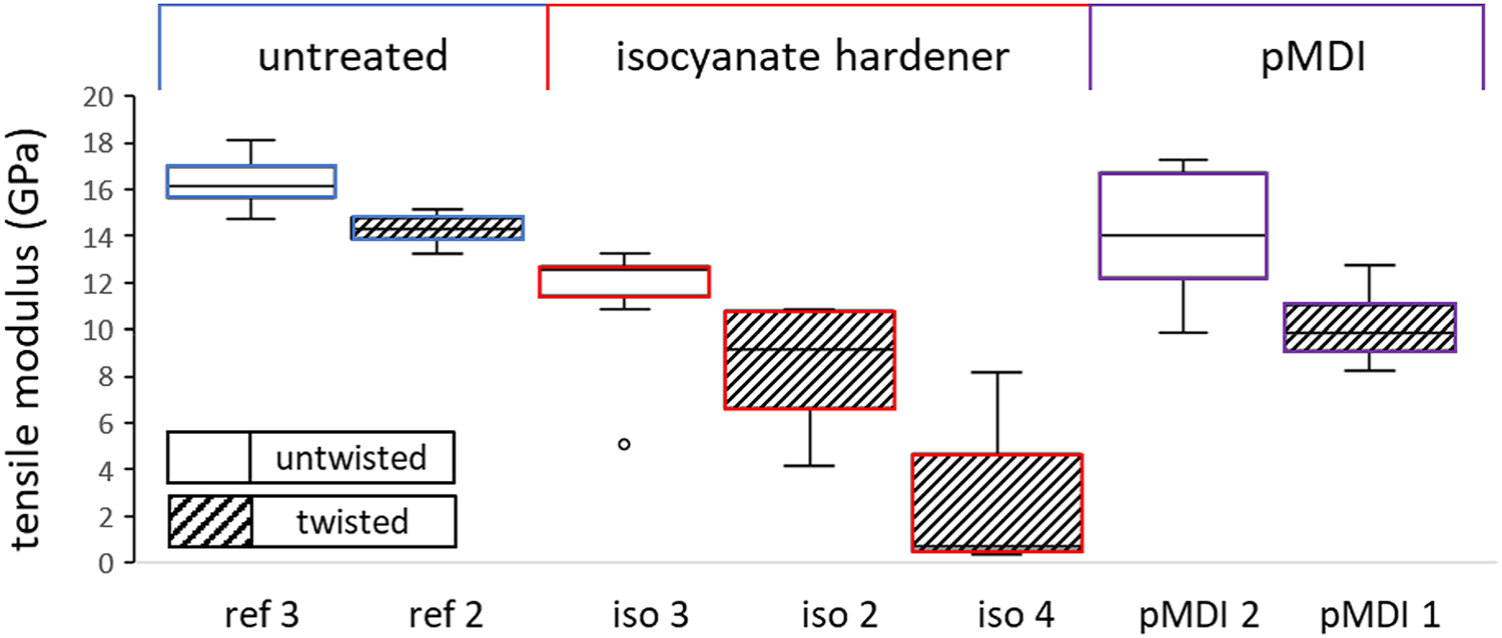
Tensile modulus of twisted and untwisted 2440 dtex samples of untreated (ref) and isocyanate-treated (iso, pMDI) yarns; isocyanate batch with 15 days treatment was additionally indicated (iso 4).

Following the theory of physical effects, a more or less brittle matrix may restrain the embedded yarn’s ability to align alongside the loading direction, thereby leading to a decrease in maximum load and elongation at break. Figure 6 compiles the tensile modulus values of untreated and treated 2440 dtex samples, providing more information on how the sample stiffness is affected. As the comparison between reference, isocyanate-hardener and pMDI batches reveals, tensile modulus decreased significantly as well for all treatments applied. Even for untwisted pMDI yarns (pMDI 2), the batch coming closest to the reference, the t-test verified a significant mean value difference, at a p value of 0.015.

Again, the drop in performance is more pronounced for twisted yarns, but the fact that a significant decrease occurred for untwisted samples too must be emphasised; adding matrix compounds around straight-oriented filaments should lead to an increase in or at least balance the tensile modulus. A decrease in tensile stiffness only could be generated as a consequence of damage within the cellulose chains. These negative effects on maximum load, elongation at break and tensile modulus may be related to chemical interactions between regenerated cellulose hydroxyl groups and matrix isocyanate groups or to the interpenetration of single filaments by the matrix, disturbing the network of cellulose fibrils.

### Mechanical characterisation of individual viscose filaments

**Figure 7:**
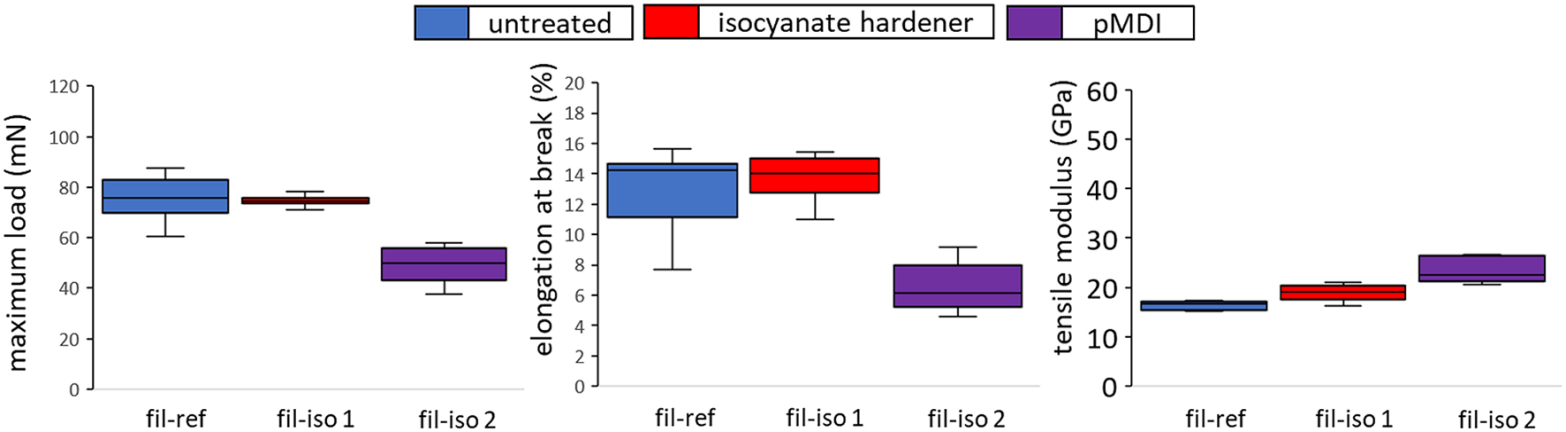
Tensile properties of single viscose filaments without further treatment (fil-ref) and treated with isocyanate hardener for 10 s (fil-iso 1) and 3 days (fil-iso 2), respectively.

**Figure 8:**
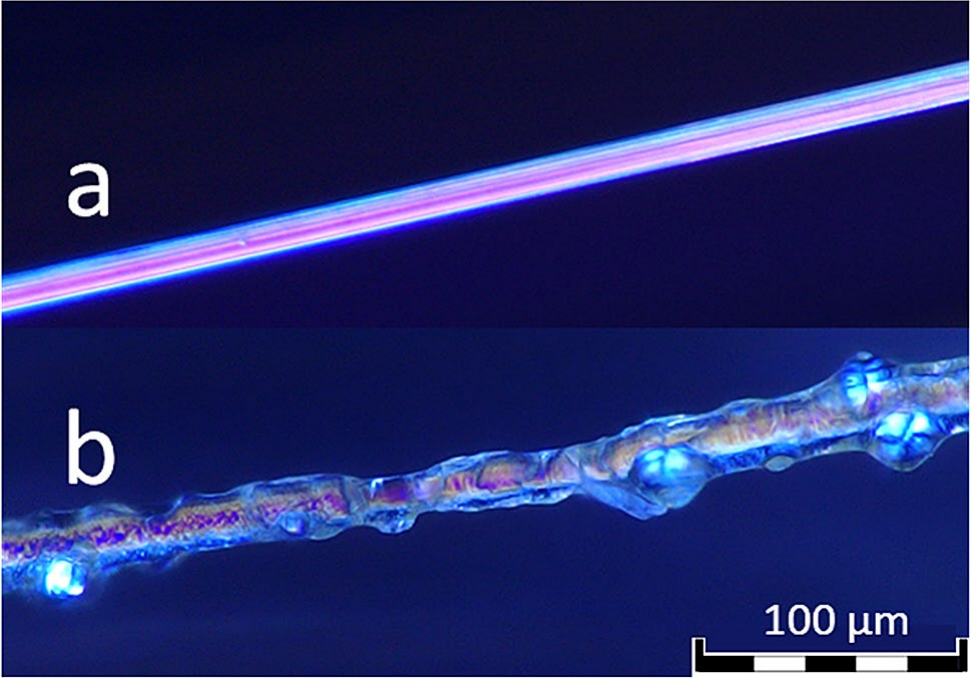
Digital microscope scan of an untreated viscose filament (**a**) and a filament subjected to 3 days immersion in isocyanate hardener (**b**), observation method: PO (polarization), objective lens: DSX10-XLOB20X, zoom: ×2.5, total magnification: ×700.

Single filament tensile tests were conducted to ensure that these observations were not just a matter of macroscopic phenomena related to the yarn structure (see Fig. 7). Single filaments from a 2440 dtex yarn were subjected to two different treatments with isocyanate hardener: either short dipping for approximately 10 s or three days of immersion in the hardener bath prior to drying under room temperature. Short-dipped samples did not show any remarkable differences from untreated reference samples. On these samples, however, almost no hardener remained on the filament surface, which could be explained by the difference in polarity. For those samples immersed for 3 days, a film of isocyanate remained on the surface, as microscopic scans show (see Fig. 8), which possibly indicates a polymerisation process on the cellulosic surface. The resulting increase in cross-sectional area could be an explanation for the higher tensile modulus values compared to untreated viscose. Regarding tensile force and elongation at break, isocyanate samples treated for three days showed significant losses, as did the treated yarn samples, confirming the observations on the level of entire yarns.

### Effect of treatment conditions

**Figure 9:**
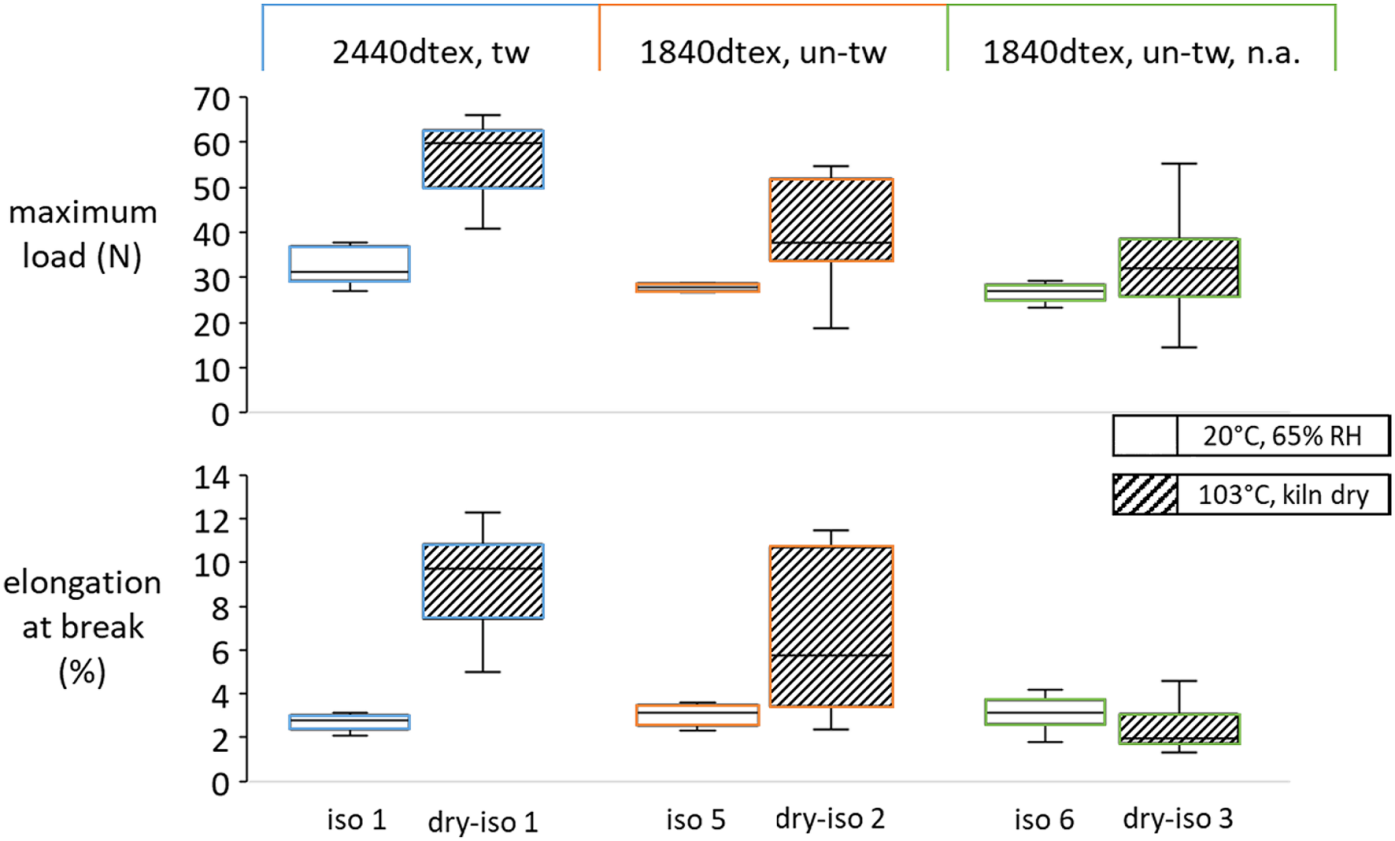
Tensile properties of twisted (tw) and untwisted (un-tw) yarns with and without (n.a.) avivage, respectively, conditioned at 20 °C, 65% RH or kiln dried at 103 °C prior to impregnation with isocyanate hardener.

Since moisture has a crucial impact on both the properties of viscose and the curing behaviour of isocyanates, it was considered as a testing parameter for the sample preparation. At the moment of dipping into isocyanate hardener, yarns had been climatised to 20 °C, 65% RH or 103 °C kiln-dried. The results in Fig. 9 show significant differences between the moisture content of kiln-dried and standard-climate yarns of approximately 0% and approximately 12%, respectively^15^. Kiln-dried samples exhibited higher variation but also significantly higher values for tensile force and elongation at break, except for the yarn batch without avivage. Whereas moist samples treated with isocyanate attained more or less the same (low) values for all three batches, dry samples showed a higher dependency on the yarn type. For instance, dry 2440 dtex samples performed better than 1840 dtex ones, as they contained more filaments per yarn. The same applied to batches with equal twist (see iso3/ iso5 in Table 1). It seems, therefore, that a higher internal water content intensifies interactions between isocyanates and viscose, which obviously have a detrimental effect on mechanical yarn properties.

Moreover, no difference could be observed between the batches with and without avivage under standard conditions (iso 5 and iso 6). The large differences between kiln-dried batches dry-iso 1 and dry-iso 2 could be ascribed to the different amount of matrix (77.3% and 53%), rather than the presence of an avivage.

### Analysis of chemical interactions

**Figure 10:**
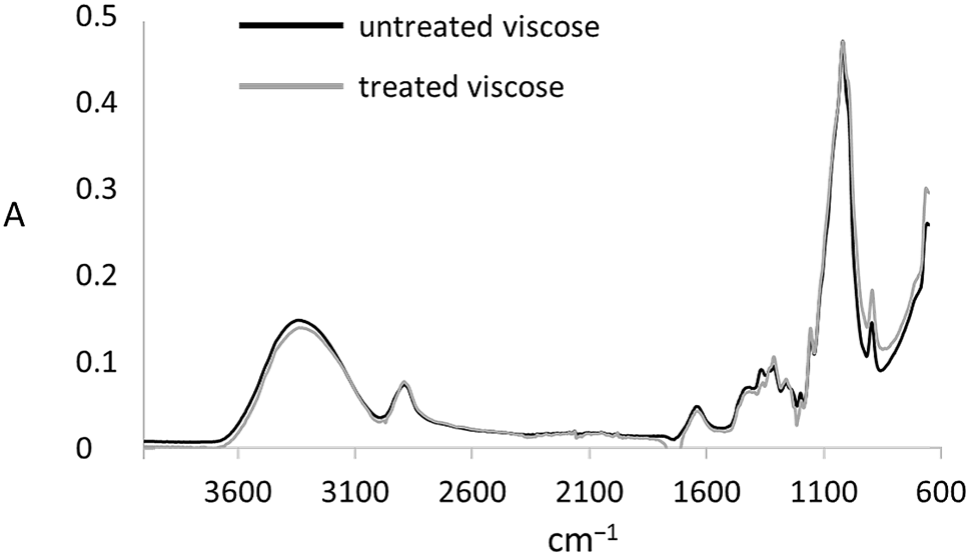
IR spectra of untreated (black line) and pMDI-hardener treated and NMP purified (grey line) yarns.

Besides the physical effects discussed, chemical interactions between regenerated cellulose and isocyanate compounds may equally the cause of inferior mechanical performance, an example being the formation of carbamate groups through crosslinking of hydroxyls of cellulose and isocyanate–NCO groups. Although, for natural fibre-based composites, various authors describe positive mechanical effects on the use of isocyanate matrices^30–34^, pure cellulose filaments with comparatively low molecular weight may end up with more brittle and fragile structures. The presence of forming covalent links was examined via IR-spectroscopy measurements. Comparisons between the spectra of untreated and treated/NMP-purified yarns (Fig. 10) shows clear evidence that no covalent modifications were present. Neither the characteristic stretching band of isocyanate groups at 2266.cm, nor that of carbamate groups at 1506/cm (C–N stretching and N–H bending of amide II bands) and 1230/cm (C–N stretching and N–H bending of amide III bands)^35^ appeared on the spectrum of the treated yarns.

If the mechanical properties of those NMP-purified samples had also shown results similar to untreated viscose yarns, then physical fibre–matrix effects (yarn twist, waviness, etc.) or curing phenomena would serve to explain the decrease in mechanical performance. As the results from single-filament tensile tests show, this is clearly not the case. The maximum load dropped around a third to 48.7 ± 8.9 mN for treated (fil-iso 3) compared to reference (fil-ref) filaments.

**Figure 11:**
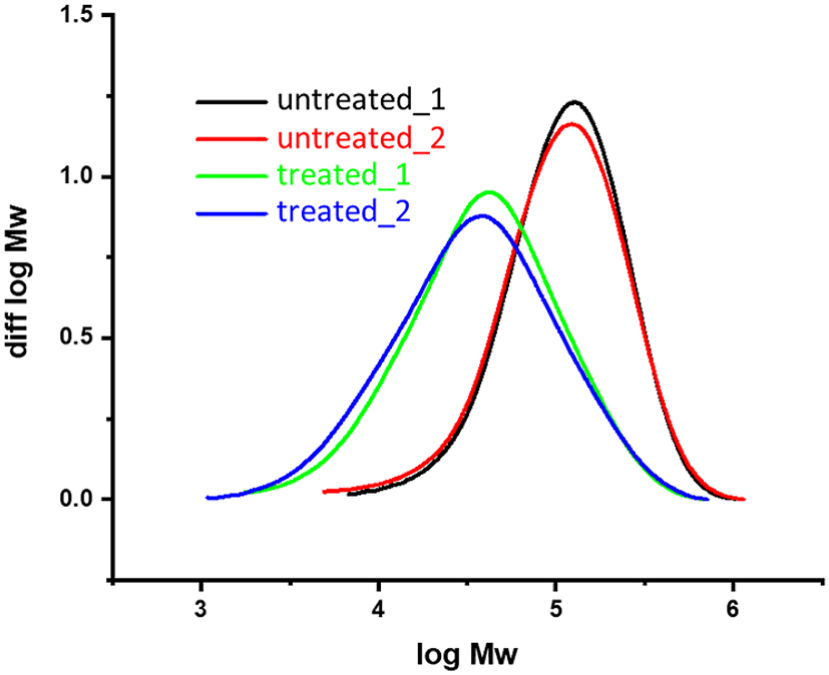
molar mass distribution of 2440 dtex yarns without treatment (black and red line) and with pMDI-hardener treatment followed by purification with NMP (green and blue line).

Another reason for inferior mechanical performance could be a decrease in the average molar mass of cellulose molecules. Hence, the samples were analysed by SEC–MALLS, a method providing absolute data on molar mass distribution for cellulose. The data are summarised in Table 2 and the molar mass distribution is presented in Fig. 11. Data clearly show a very strong degradation of molar mass and a significant broadening of the distribution, i.e. a higher dispersity. The degradation pattern indicates a hydrolytic process. Based on the origin of an acid as a possible cause, the reagent itself has to be considered, as hydrochloric acid is a common by-product of pMDI synthesis^23^.

**Table 2:** Molar mass information for each pair of untreated and treated samples.

Viscose 2440 dtex	Mn (kDa)	Mw (kDa)	Mz (kDa)	Dispersity Ɖ (Mw/Mn)
Untreated 1	80.53	144.9	225.9	1.799
Untreated 2	71.38	143.1	234.2	2.005
Treated 1	23.07	61.06	130.4	2.646
Treated 2	19.6	58.56	136	2.987

For the pMDI purchased from SIGMA-ALDRICH, the certificate of analysis states 0.0021% of residual HCl. This may seem a low concentration, but in combination with an organic environment and extended reaction time, it can be highly detrimental to cellulose integrity. To test further whether the acid caused the effects observed, the second batch of filament samples (fil 5) was treated with an isocyanate bath containing calcium carbonate to neutralise the HCl present. A comparison of the respective tensile tests from batches fil-iso 3 and fil-iso 4 (see Table 1) clearly indicated improved load and elongation properties for those samples in the presence of calcium carbonate. This underlines on the one hand, the importance of the process parameter acidity in the context of composites containing cellulose filaments and, on the other hand, offers a simple means of avoiding damage to reinforcing fibres. Nevertheless, the maximum load properties of calcium carbonate-blended filaments averaged around 15% below the level of the untreated filaments. Further investigations are needed to clarify whether an increased amount of neutralisation agent helps improve properties further.

## Conclusions

In order to find new applications for cellulose-based materials in high-performance composite structures, a systematic analysis of fibre matrix interactions was conducted. Process parameters describing these interactions were detected through a combination of chemical analysis and mechanical tests at the level of yarns and single filaments.

It was found, by means of tensile tests that treatments with pMDI and a pMDI-based hardener significantly decreased the maximum load, elongation at break and tensile modulus of viscose yarns. Since these observations also applied to single filament-tests, it may be deduced that an irreversible modification of the cellulosic microstructure occurred.

Isocyanate-treated samples with higher yarn twist were found to perform worse than untwisted ones. This phenomenon did not occur in untreated samples, rendering yarn twist an essential structural parameter for fibre–matrix composites. With regard to chemical parameters, enhanced matrix acidity appeared to decrease cellulose molar mass significantly and destabilise the filament’s internal structure. Apart from chemical and structural aspects, cellulose filament moisture content was found to be a third fundamental parameter affecting the composite performance, with higher water content enhancing the strength-reducing effects of the isocyanate matrix.

From these results, the application of isocyanate-based systems for regenerated cellulose composites appears, at first sight, to be a challenge. With sufficient knowledge about critical process parameters, however, negative constellations can be avoided while taking advantage of versatile possibilities for cellulose modifications towards bio-based, high-performance composites.

## Data Availability

All data presented in this study has been submitted in the manuscript.

## References

[1] Reddy MM, Vivekanandhan S, Misra M, Bhatia SK, Mohanty AK (2013). Biobased plastics and bionanocomposites: Current status and future opportunities. Prog. Polym. Sci..

[2] Helanto K, Matikainen L, Talja R, Rojas OJ (2019). Bio-based polymers for sustainable packaging and biobarriers: A critical review. BioResources.

[3] Kohl D, Link P, Böhm S (2016). Wood as a technical material for structural vehicle components. Procedia CIRP.

[4] Berthold, D. Holzformteile als multi-materialsysteme für den Einsatz im Fahrzeug-Rohbau (HAMMER). Fraunhofer- Institut für Holzforschung, Wilhelm-Klauditz-Institut (WKI) 10.2314/GBV:874862787 (2016).

[5] Müller U, Jost T, Kurzböck C, Stadlmann A, Wagner W, Kirschbichler S, Baumann G, Pramreiter M, Feist F (2019). Crash simulation of wood and composite wood for future automotive engineering. Wood Mater. Sci. Eng..

[6] Chawla, K. K. Reinforcements. In *Composite Materials: Science and Engineering* (ed. Chawla, K.K.) 7–71. (Springer New York, 2012)

[7] El-Dessouky, H. M. Spread Tow Technology for Ultra Lightweight CFRP Composites: Potential and Possibilities. In *Advanced composite materials: Properties and applications.*(ed. Bafekrpour, E.) 323–348 (De Gruyter, 2017)

[8] Friedrich K, Almajid AA (2013). Manufacturing aspects of advanced polymer composites for automotive applications. Appl. Compos. Mater..

[9] Cherif C (2011). Textile Werkstoffe für den Leichtbau.

[10] Rana, S., Pichandi, S., Parveen, S., Fangueiro, R. Regenerated Cellulosic Fibers and Their Implications on Sustainability. In *Roadmap to Sustainable Textiles and Clothing* (ed. Muthu, S.S.) 239–276. (Springer, 2014)

[11] Kaserer, W., Winkelmeier, D. *Annual report 2018* (ed. Kaserer, W., Winkelmeier, D.) 26-52 (Lenzing Aktiengesellschaft, 2019) https://www.lenzing.com/de/investoren/publikationen.

[12] Gibson LJ (2012). The hierarchical structure and mechanics of plant materials. J. R. Soc. Interface.

[13] Shah DU (2014). Natural fibre composites: Comprehensive Ashby-type materials selection charts. Mater. Des..

[14] Reusch, W. Reactions of alcohols. In *Virtual Textbook of Organic Chemistry* (ed. Reusch, W.) https://www2.chemistry.msu.edu/faculty/reusch/VirtTxtJml/alcohol1.htm#alcnom. (2013).

[15] Siroka B, Noisternig M, Griesser UJ, Bechtold T (2008). Characterization of cellulosic fibers and fabrics by sorption/desorption. Carbohyd. Res..

[16] Six, C. & Richter, F. Isocyanates, Organic. In *Ullmann's encyclopedia of industrial chemistry*. (eds. Arpe, H., Ullmann, F.) (Wiley-VCH, 2003).

[17] Frihart CR, Rowell RM (2005). Wood adhesion and adhesives. Handbook of Wood Chemistry and Wood Composites.

[18] Hill, C. Chemical modification of wood (II): Reaction with other chemicals. In *Wood Modification* (ed. Stevens, C.) 77–97. (Wiley, 2006).

[19] Rowell RM, Ellis WD (1984). Effects of moisture on the chemical modification of wood with epoxides and isocyanates. Wood Fiber Sci..

[20] Yelle D, Ralph J, Frihart C (2011). Delineating pMDI model reactions with loblolly pine via solution-state NMR spectroscopy. Part 2. Non-catalyzed reactions with the wood cell wall. Holzforschung.

[21] Marcinko JJ, Rinaldi PL, Bao S (1999). Exploring the physicochemical nature of PMDI/wood structural composite adhesion. For. Prod. J..

[22] Frazier CE, Ni J (1998). On the occurrence of network interpenetration in the wood-isocyanate adhesive interphase. Int. J. Adhes. Adhes..

[23] Mortimer CE, Müller U, Müller U (2010). Organische Chemie, Teil II: Funktionelle Gruppen. Chemie.

[24] Adusumali R-B, Reifferscheid M, Weber H, Röder T, Sixta H, Gindl W (2006). Mechanical properties of regenerated cellulose fibres for composites. Macromol. Symp..

[25] Adusumalli R-B, Müller U, Weber H, Roeder T, Sixta H, Gindl W (2006). Tensile testing of single regenerated cellulose fibres. Macromol. Symp..

[26] Kim I-C, Kim J-H, Lee K-H, Tak T-M (2002). Preparation of soluble copolyurethaneimide containing oxyethylene and oxypropylene units. J. Appl. Polym. Sci..

[27] Potthast A, Radosta S, Saake B, Lebioda S, Heinze T, Henniges U, Isogai A, Koschella A, Kosma P, Rosenau T, Schiehser S, Sixta H, Strlič M, Strobin G, Vorwerg W, Wetzel H (2015). Comparison testing of methods for gel permeation chromatography of cellulose: Coming closer to a standard protocol. Cellulose.

[28] Zhou X, Frazier CE (2001). Double labeled isocyanate resins for the solid-state NMR detection of urethane linkages to wood. Int. J. Adhes. Adhes..

[29] Chun H-J, Shin J-Y, Daniel IM (2001). Effects of material and geometric nonlinearities on the tensile and compressive behavior of composite materials with fiber waviness. Compos. Sci. Technol..

[30] Huang G, Wang P (2017). Effects of preparation conditions on properties of rigid polyurethane foam composites based on liquefied bagasse and jute fibre. Polym. Testing.

[31] Bakare IO, Okieimen FE, Pavithran C, Khalil HPSA, Brahmakumar M (2010). Mechanical and thermal properties of sisal fiber-reinforced rubber seed oil-based polyurethane composites. Mater. Design.

[32] Merlini C, Soldi V, Barra GMO (2011). Influence of fiber surface treatment and length on physico-chemical properties of short random banana fiber-reinforced castor oil polyurethane composites. Polym. Testing.

[33] Jiang L, Chen F, Qian J, Huang J, Wolcott M, Liu L, Zhang J (2010). Reinforcing and Toughening Effects of Bamboo Pulp Fiber on Poly(3-hydroxybutyrate-co-3-hydroxyvalerate) fiber composites. Ind. Eng. Chem. Res..

[34] Huang G, Chen F (2019). Reaction of jute fiber with isocyanate component for the production of plant fiber-reinforced polyurethane composites. Cellulose.

[35] Luo W (1997). Ying: hydrogen-bonding properties of segmented polyether poly(urethane urea) copolymer. Macromolecules.

